# Near‐normal ultrasound morphology in quadriceps tendon after anterior cruciate ligament graft harvest

**DOI:** 10.1002/jeo2.70463

**Published:** 2025-10-28

**Authors:** Jumpei Inoue, Kohei Kamada, Keishi Takaba, M. Enes Kayaalp, Joseph D. Giusto, Bryson P. Lesniak, Andrew L. Sprague, Volker Musahl

**Affiliations:** ^1^ UPMC Freddie Fu Sports Medicine Center, Department of Orthopaedic Surgery University of Pittsburgh Medical Center Pittsburgh Pennsylvania USA; ^2^ Department of Orthopaedic Surgery University of Pittsburgh Pittsburgh Pennsylvania USA; ^3^ Department of Orthopaedic Surgery Nagoya Tokushukai General Hospital Kasugai Aichi Japan; ^4^ Department of Orthopaedic Surgery Kobe University Graduate School of Medicine Kobe Hyogo Japan; ^5^ Department for Orthopaedics and Traumatology University of Health Sciences, Istanbul Fatih Sultan Mehmet Training and Research Hospital Istanbul Turkey; ^6^ Department of Physical Therapy, School of Health and Rehabilitation Sciences University of Pittsburgh Pittsburgh Pennsylvania USA

**Keywords:** anterior cruciate ligament reconstruction, graft survival, quadriceps muscle, tendons/diagnostic imaging, ultrasonography

## Abstract

**Purpose:**

The quadriceps tendon (QT) autograft is increasingly used for anterior cruciate ligament reconstruction (ACLR) due to favourable clinical outcomes. However, the long‐term morphological and vascular changes at the QT harvest site remain unclear. This study aimed to evaluate QT morphology and vascularity using ultrasound at least 1 year after ACLR.

**Methods:**

Thirty‐five patients who underwent ACLR with a QT autograft were prospectively evaluated at a mean of 3.4 ± 3.5 years post‐operatively. Surgical technique was not standardized with respect to harvest thickness or bone block inclusion. Ultrasound was used to assess anterior‐to‐posterior (A‐P) thickness, medial‐to‐lateral (M‐L) width, cross‐sectional area (CSA) and echogenicity of the QT in both knees at 15, 30, 45 and 60 mm proximal to the patella. Superb microvascular imaging (SMI) was employed to evaluate vascularity at the same locations. Comparisons were made between the operated and contralateral sides.

**Results:**

A‐P thickness was significantly greater in the operated knee at 30 mm (by 0.7 mm, *p* = 0.01), 45 mm (by 1.1 mm, *p* < 0.01) and 60 mm (by 1.3 mm, *p* < 0.01) proximal to the patella. No significant differences were found in M‐L width or CSA. Echogenicity was significantly lower in the harvested QT at all levels (*p* < 0.01), potentially indicating altered tendon quality. Vascularity was observed in 28.6% of patients, primarily within 2 years post‐operatively (*p* = 0.02). A defect at the harvest site was identified in 20% of patients, with a mean size of 16.1% of the total QT CSA. No post‐operative QT ruptures or wound complications were reported.

**Conclusion:**

QT morphology generally normalized by 3.4 years post‐ACLR, although reduced echogenicity persisted, suggesting potential changes in tendon quality. Despite these findings, no clinical complications were observed, supporting the long‐term safety of QT autograft harvest for ACLR.

**Level of Evidence:**

Level IV.

**Abbreviations**
ACL: anterior cruciate ligament
ACLR: anterior cruciate ligament reconstruction
AP: anterior‐posterior
CSA: cross‐sectional area
ICC: intraclass correlation coefficient
ML: medial‐lateral
MRI: magnetic resonance imaging
QT: quadriceps tendon
SEM: standard error of measurement
SMI: superb microvascular imaging

## INTRODUCTION

The quadriceps tendon (QT) autograft is increasingly used for anterior cruciate ligament (ACL) reconstruction (ACLR) [1, 6]. The growing number of platforms providing information about QT ACLR reflects the rising interest in this graft option [7]. Benefits of using a QT autograft include comparable or lower retear rates and reduced harvest site pain compared to other options, such as hamstring and patellar tendon autografts [8, 20, 21, 27]. However, quadriceps muscle recovery may be delayed following QT autograft harvest compared to hamstring tendon harvest [11].

Despite concern among both patients and surgeons regarding donor‐site healing, there is limited information on the morphological changes of the QT after autograft harvest [5, 12]. One magnetic resonance imaging (MRI) study showed near‐complete QT healing 2 years post‐ACLR, but this was limited to the distal 30 mm of the tendon [12]. A better understanding of tendon healing is important for setting patient expectations and guiding surgical decision‐making when selecting graft options.

Ultrasound assessment, specifically through superb microvascular imaging (SMI), has been utilized to evaluate the healing response of tendons by measuring their vascularity [2, 10, 23, 28]. SMI is capable of detecting low‐velocity, low‐volume blood flow with greater sensitivity than conventional Doppler imaging. A recent study using SMI reported increased vascularity in the QT for up to 1 year after harvest [22]. However, data on post‐operative vascularity beyond 1 year remain limited. Persistent hypervascularity beyond this period may indicate ongoing chronic inflammation rather than a normal healing response.

The purpose of this study was to evaluate the post‐operative morphological and vascular changes throughout the entire QT harvest site at a minimum of 1 year following ACLR. It was hypothesized that the post‐operative QT size, including anterior‐posterior (AP) thickness, medial‐lateral (ML) width and cross‐sectional area (CSA), as well as vascularity, would not differ from that of the contralateral side 1 year after ACLR.

## MATERIALS AND METHODS

Thirty‐five patients were prospectively recruited for the study and provided institutional review board‐approved written informed consent prior to any research procedures. Inclusion criteria were as follows: (1) patients who underwent primary or revision ACLR with a QT autograft, (2) were at least 1 year post‐ACLR and (3) were 14 years of age or older at the time of ACLR. Exclusion criteria included: (1) multi‐ligament surgery and (2) bilateral ACLR with a QT autograft.

### Ultrasonographic QT assessment

Ultrasonographic examinations were performed on both the operated and contralateral knees by an orthopaedic surgeon (J.I.), who has more than 10 years of experience in ultrasound assessments. The ultrasound settings and patient positioning used for the assessment were previously described in previous studies [13, 25]. An 18‐5 MHz linear‐array transducer (Aplio i800; Canon Medical Systems) was employed to assess participants positioned supine with the knee flexed at 20°. The transducer was placed anteriorly to the knee, perpendicular to the tendon fibres of the QT and adjusted to capture an image parallel to the widest diameter of the QT. The depth, frequency and gain were set at 20–30 mm (depending on the patient), 17 MHz and 80 dB, respectively. Measurement sites at 15, 30, 45 and 60 mm proximal to the superior pole of the patella were marked on the skin with a pen using a ruler.

**Figure 1:**
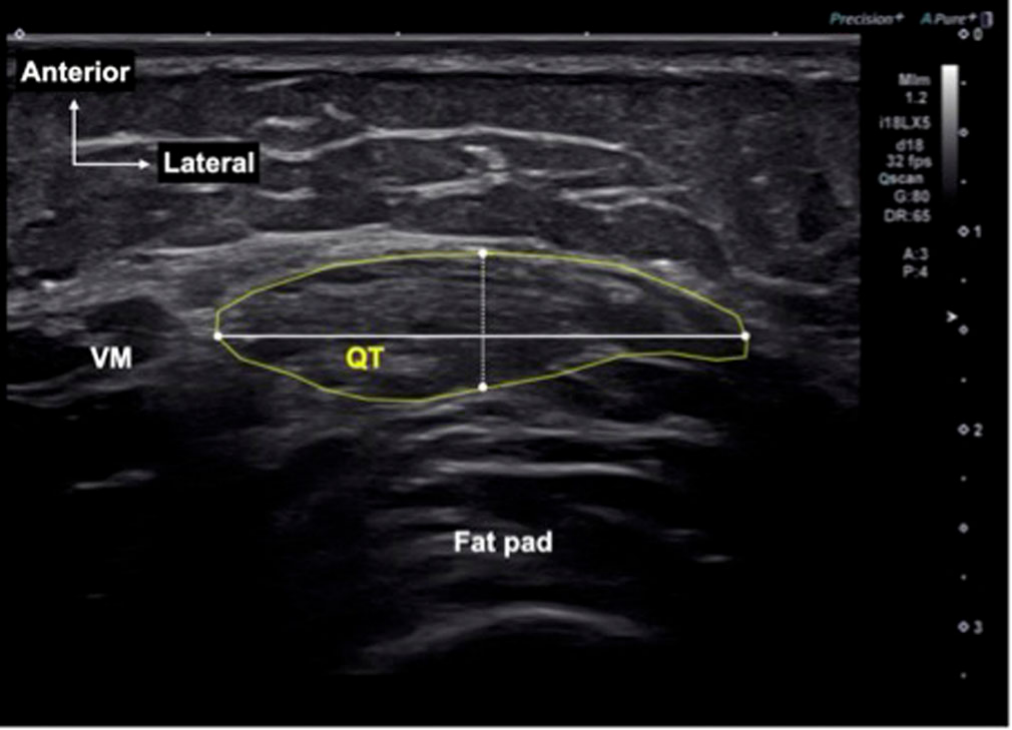
Measurement of anterior‐posterior thickness, medial‐lateral width, and cross‐sectional area (CSA) of the quadriceps tendon (QT) on magnetic resonance imaging. CSA (yellow line) was also measured at 15, 30, 45 and 60 mm proximal to the superior pole of the patella on short‐axis images. The widest medial‐lateral width (white line) was measured. The anterior‐posterior thickness (dotted line) was measured at the midpoint of the medial‐lateral width. VM, vastus medialis.

The short‐axis images of the QT were evaluated at all sites to measure A‐P thickness, M‐L width and CSA using the following methods: First, the CSA was determined by tracing the outline of the tendon. Next, the M‐L width of the QT was measured at its widest diameter parallel to the tendon (Figure 1). Finally, the A‐P thickness was measured at the midpoint of the line drawn to determine the M‐L width. The measurements were performed using ImageJ software (National Institutes of Health).

**Figure 2:**
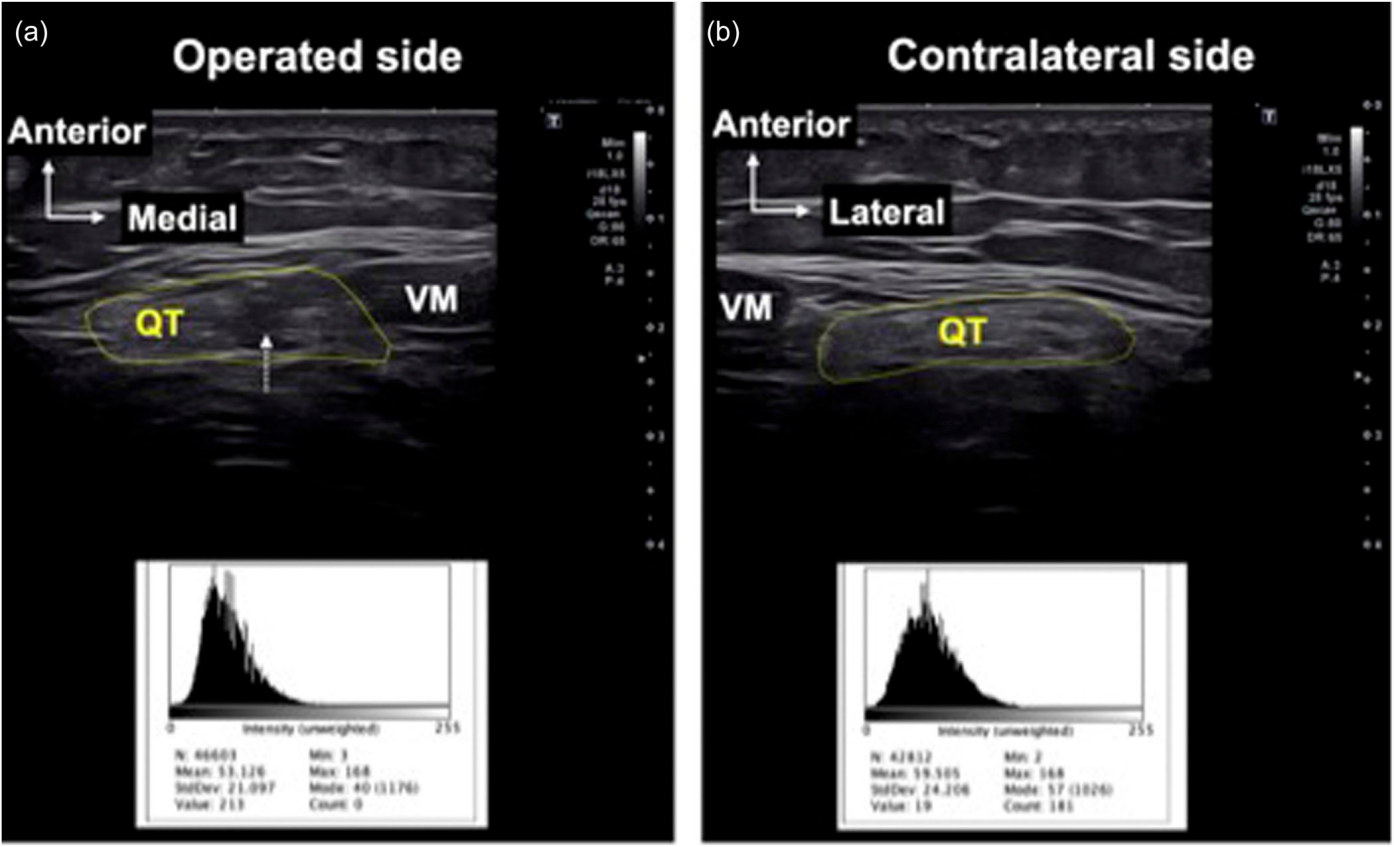
Echogenicity measurement of the quadriceps tendon (QT). Echogenicity was assessed using ImageJ software, which measured the intensity within the region of interest (yellow line), with the whitest intensity set at 255 and the blackest intensity at 0. Echogenicity was defined as the mean grayscale value. (a) Operated side: a defect is visible at the midpoint of the tendon (white dotted arrow). Echogenicity measured 53.1. (b) Contralateral side: Echogenicity measured 59.5. VM, vastus medialis.

The echogenicity was evaluated at 15, 30, 45 and 60 mm proximal to the superior pole of the patella in both knees using ImageJ software (National Institutes of Health) following the methodology of a previous study [14]. Echogenicity for the entire QT was also measured in the transverse plane and defined as the mean grayscale value. In ImageJ, white intensity was set at 255 and black intensity at 0 (Figure 2). Scar tissue, which appears as darker grayscale compared to normal tendon, is expected to show lower mean echogenicity.

**Figure 3:**
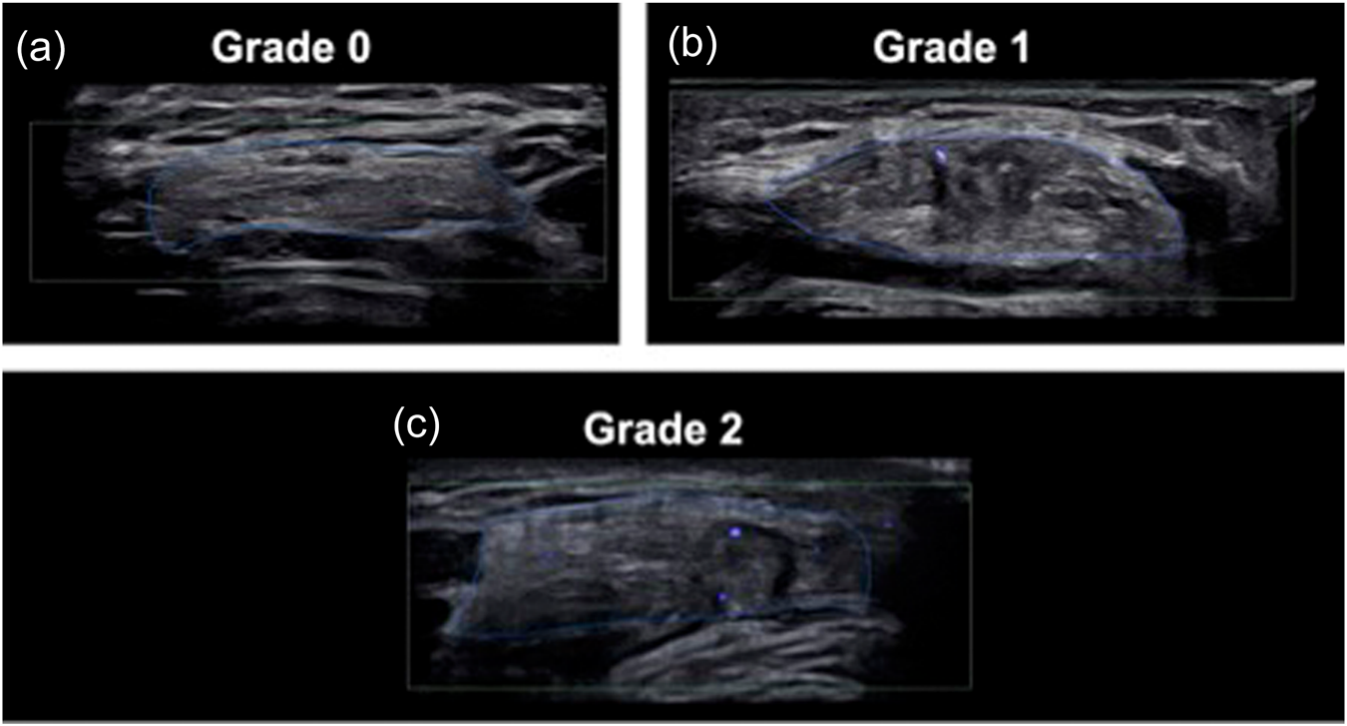
Evaluation of quadriceps tendon vascularity using superb microvascular imaging. The tendon outline is marked by a blue line. (a) Grade 0: No vascular signal. Ultrasound performed 2 years post‐operatively. (b) Grade 1: 1–2 spots of vascular signal (≤2 mm). Ultrasound performed 1 year post‐operatively. (c) Grade 2: More than 2 spots of vascular signal. Ultrasound performed 1 year post‐operatively.

**Figure 4:**
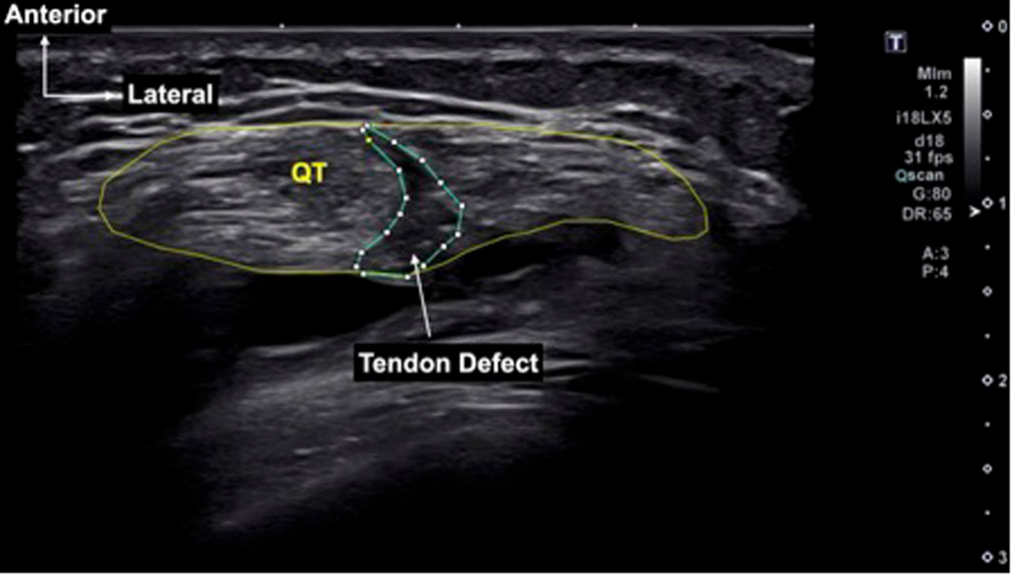
Measurement of a defect in the quadriceps tendon (QT). The defect is outlined in blue.

The vascularity of the QT was assessed using SMI at 15, 30, 45 and 60 mm proximal to the superior pole of the patella in both knees. During SMI, the vascularity scale and colour gain were set to 1.0–1.5 m/s and 35 dB, respectively, to minimize noise. A 3‐s video was recorded to further assess the vascularity signal, and the image displaying the greatest vascularity signal was selected from the acquired video. A semi‐quantitative grading scale, adapted from a previous study on ACL vascularization using SMI [26], was used for QT assessment. Vascularity signals were classified into four grades: grade 0, no vascular signal; Grade 1, 1–2 spots (shorter than 2 mm) of vascular signal; Grade 2, more than 2 spots of vascular signal (Figure 3). The intra‐ and inter‐observer agreement for vascularity grading in the previous study demonstrated a kappa value ranging from 0.85 to 0.96 [26]. Additionally, the presence of a hypoechoic defect at the harvest site was investigated. If a defect was present, its size was measured as the CSA (Figure 4). Palpation tenderness at the harvest site was assessed during the ultrasound examination.

### Surgical technique

All ACLR with QT autograft was performed by one of four high‐volume, sports fellowship‐trained orthopaedic surgeons. The protocol for QT autograft harvest, including decisions on graft diameter, partial versus full‐thickness and the inclusion of a patellar bone block, was determined at the discretion of the operating surgeon. Following graft extraction, the QT autograft harvest site was closed with sutures in all cases. Data on graft thickness (partial or full) and the presence of a patellar bone block in the harvested tendon were obtained from the surgical records.

### Statistical analysis

To calculate intra‐ and inter‐observer reliability for the measurements for A‐P thickness, M‐L width, CSA and echogenicity of QT, measurements were independently performed by 2 observers and repeated by 1 of the observers for 10 randomly selected patients. The inter‐ and intra‐observer reliabilities for the above measurement were evaluated with the intraclass correlation coefficient (ICC_2,1_ and ICC_3,1_, respectively). ICC < 0.50 was considered poor reliability, 0.50–0.75 moderate, 0.76–0.90 good and >0.90 excellent according to a previous study [17]. The standard error of measurement (SEM) was calculated using the formula: SEM = SD × √(1 − ICC). For vascularity grading, inter‐ and intra‐observer agreement was assessed by having two observers independently grade vascularity and one observer repeat the grading for 10 randomly selected patients; agreement was evaluated using the kappa statistic. Agreement was interpreted as poor (<0.00), slight (0.00–0.20), fair (0.21–0.40), moderate (0.41–0.60), substantial (0.61–0.80) and almost perfect (>0.80) [18].

The Shapiro–Wilk normality test was used to assess whether the sample followed a normal distribution. A paired *t* test was applied to compare the A‐P thickness, M‐L width, CSA and echogenicity between the operated and contralateral sides, as the data were normally distributed. Categorical variables were compared using the chi‐square test. To assess differences in morphology and echogenicity between full‐ and partial‐thickness harvests, the percentage difference from the contralateral side—calculated as ((operated side value − contralateral side value)/contralateral side value × 100)—was analyzed using independent *t* tests, based on normally distributed data. All statistical analyses were performed using SPSS software version 28.0.1.1 (IBM), with statistical significance set at *p* < 0.05.

The required sample size was determined via an a priori power analysis using G*Power v3.1.9 (Heinrich Heine University). Based on a previous study on the morphological changes of the QT after harvesting using MRI [12], at least 27 cases were needed to achieve a power of 0.80, an *α* of 0.05 and an effect size of 0.57.

## RESULTS

A total of 35 patients (mean age 26.3 ± 9.1 years, 57.1% (*n* = 20) female) were included in the final analysis. The mean height, body weight, and body mass index of the cohort were 169.6 ± 9.3 cm, 76.0 ± 15.7 kg and 26.2 ± 4.6 kg/m^2^, respectively. The mean duration between QT autograft ACLR and post‐operative ultrasound evaluation was 3.4 ± 3.5 years. The QT was harvested as a full‐thickness graft in seven (20.0%) patients, and a patellar bone block was included in the graft in three (8.6%) patients (Table 1). No palpation tenderness was noted at the harvest site during ultrasound examination in any of the patients. Details on intra‐ and inter‐observer reliability, as well as the SEM for the measurements, are shown in Table 2. The mean ICC showed good to excellent reliability for all measurements. The kappa values for vascularity grading indicated almost perfect agreement, with values of 0.824 for inter‐observer and 0.918 for intra‐observer reliability.

**Table 1:** Patient characteristics.

Variable	Value
Age, years, mean ± SD	26.3 ± 9.1
Female, *n* (%)	20 (57.1%)
Right side, *n* (%)	16 (45.7%)
Height, cm, mean ± SD	169.6 ± 9.3
Body weight, kg, mean ± SD	76.0 ± 15.7
Body mass index, kg/m^2^, mean ± SD	26.2 ± 4.6
Duration between post‐operative ultrasound and surgery, years, mean ± SD (range)	3.4 ± 3.5 (1.0–14.0)
Full thickness harvest, *n* (%)	7 (20%)
Existence of a patellar bone block, *n* (%)	3 (8.6%)
Revision ACLR, *n* (%)	5 (14.3%)

*Note*: Data are reported as mean ± SD (range) in the case of continuous variables or number (%) in the case of dichotomous variables.

Abbreviations: ACLR, anterior cruciate ligament reconstruction; SD, standard deviation.

**Table 2:** Intra‐ and inter‐observer reliability for ultrasound measurements.

	A‐P thickness	M‐L width	CSA	Echogenicity
ICC_3,1_	0.875 (0.812–0.918)	0.832 (0.751–0.889)	0.821 (0.734–0.881)	0.949 (0.922–0.967)
SEM	0.5 mm	1.8 mm	15.8 mm^2^	2.0
ICC_2,1_	0.856 (0.785–0.905)	0.817 (0.727–0.879)	0.849 (0.775–0.901)	0.913 (0.838–0.950)
SEM	0.5 mm	1.9 mm	14.1 mm^2^	2.9

*Note*: Data are reported as mean (95% CI).

Abbreviations: A‐P, anterior‐posterior; CI, confidence interval; CSA, cross‐sectional area; ICC, interclass correlation coefficient; M‐L, medial‐lateral.

**Figure 5:**
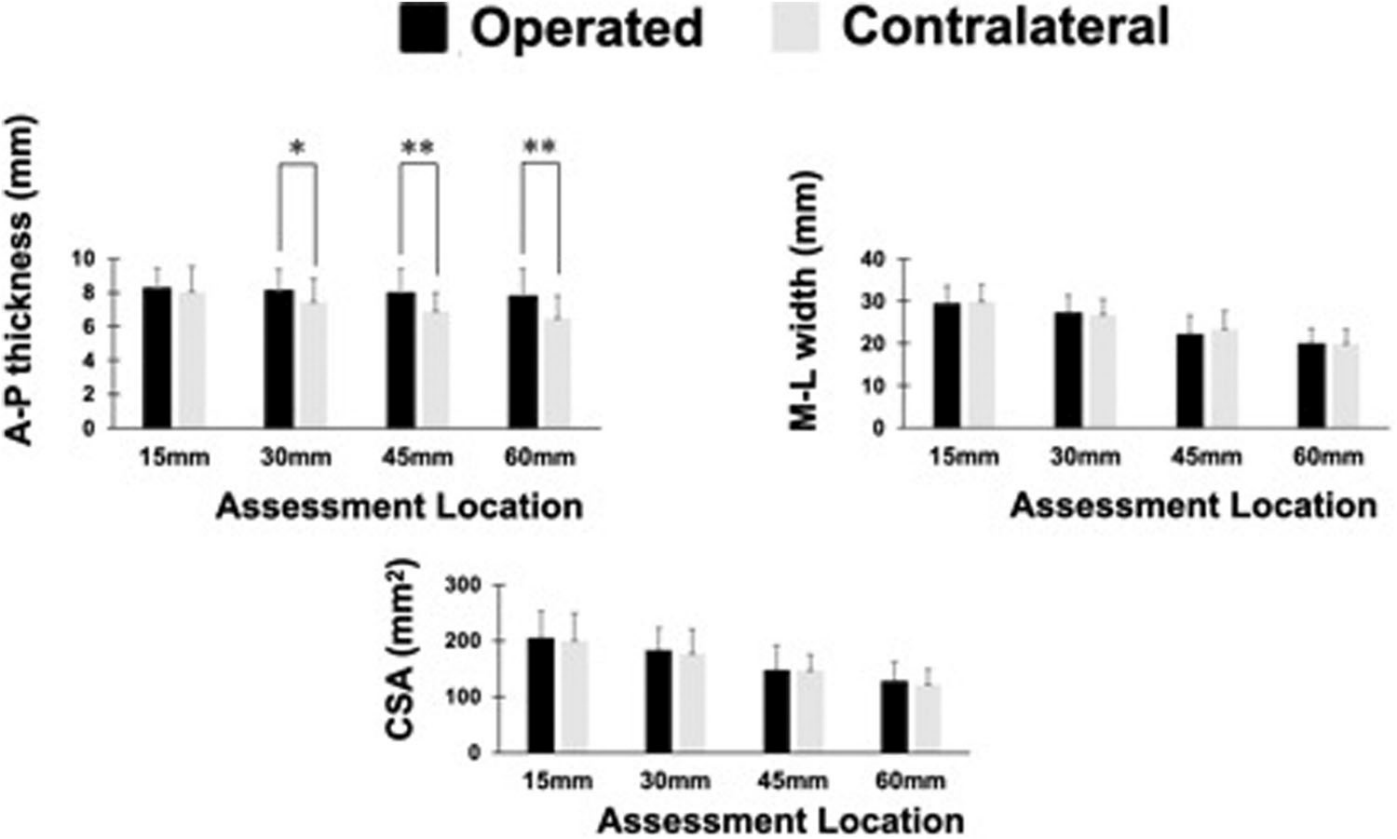
Comparison of anterior‐posterior (A‐P) thickness, medial‐lateral (M‐L) width and CSA of the quadriceps tendon between operated and contralateral knees. CSA, cross‐sectional area. *Significant differences (*p* < 0.05). **Significant differences (*p* < 0.01).

Compared to the contralateral side, the A‐P thickness of the post‐operative QT donor site was increased by 8.9% at 30 mm (7.4 ± 1.4 mm vs. 8.1 ± 1.2 mm, *p* = 0.01), 16.0% at 45 mm (6.9 ± 1.1 mm vs. 8.0 ± 1.4 mm, *p* < 0.01) and 19.9% at 60 mm proximal to the patella (6.5 ± 1.3 mm vs. 7.8 ± 1.6 mm, *p* < 0.01). All observed differences exceeded the SEM of 0.5 mm. Among patients less than 2 years post‐operatively, the A‐P thickness increased by 16.6%, 22.5% and 29.0% at 30, 45 and 60 mm, respectively. In contrast, patients more than 2 years after surgery showed increases of 4.9%, 12.5% and 14.9% at 30, 45 and 60 mm, respectively. No difference in the M‐L width and CSA of the QT at each assessment location was observed between operated and contralateral sides (Figure 5).

**Figure 6:**
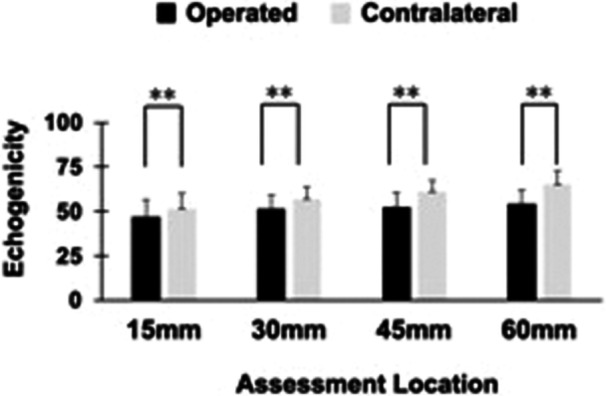
Comparison of echogenicity between operated and contralateral knees. **Significant differences (*p* < 0.01).

The echogenicity of the QT was significantly lower in the operated side at all assessment locations compared to the contralateral side (15 mm; 51.7 ± 8.3 vs. 47.0 ± 9.7, *p* < 0.01, 30 mm; 57.1 ± 6.7 vs. 51.7 ± 7.5, *p* < 0.01, 45 mm; 61.2 ± 6.2 vs. 52.3 ± 8.2, *p* < 0.01, 60 mm; 65.1 ± 7.7 vs. 54.4 ± 7.8, *p* < 0.01, Figure 6). The differences at all measured locations exceeded the SEM of 2.0. Among patients less than 2 years post‐operatively, echogenicity decreased by 11.5%, 13.6%, 23.7% and 20.6% at 15, 30, 45 and 60 mm, respectively. In contrast, patients more than 2 years after surgery showed increases of 7.6%, 7.2%, 9.7% and 14.2% at 15, 30, 45 and 60 mm, respectively, at the corresponding locations.

No vascularity was observed on the contralateral side at any measurement location, except for a single case of Grade 1 vascularity at 15 mm proximal to the patella. In contrast, vascularity was detected on the operated side at various measurement locations in 10 patients (28.6%). Notably, vascularity was less common in patients with a follow‐up of more than 2 years, with only 3 out of 23 patients (13.0%) exhibiting vascularity, compared to 7 out of 12 patients (58.3%) with a follow‐up of less than 2 years (chi‐square test, *p* = 0.02). Specifically, vascularity was present in eight patients (22.9%) at 15 mm proximal to the patella (Grade 1: five patients; Grade 2: three patients), in three patients (8.6%) at 30 mm (all Grade 1), in three patients (8.6%) at 45 mm (Grade 1: one patient; Grade 2: two patients) and in one patient (2.9%) at 60 mm (Grade 1).

A defect in the post‐operative QT was observed in seven patients (20%). Specifically, defects were seen in four patients (11.4%) at 15 mm, five patients (14.3%) at 30 mm, six patients (17.1%) at 45 mm and two patients (5.7%) at 60 mm proximal to the patella. Among patients less than 2 years post‐operatively, 4 out of 12 (33.3%) exhibited a defect, whereas 3 out of 23 (13.0%) in those more than 2 years after surgery showed a defect (*p* = n.s.). The mean size of the defect was 27.4 mm^2^, representing 16.1% of the total CSA of the QT. There were no post‐operative QT ruptures or would‐related complications.

### Full‐ versus partial‐thickness harvest

The mean duration from QT autograft ACLR to post‐operative ultrasound evaluation was 6.1 ± 5.5 years in the full‐thickness harvest group and 2.7 ± 2.6 years in the partial‐thickness group (*p* = n.s.). When comparing full and partial thickness harvests, no significant differences were observed in thickness, width or CSA between the operated and contralateral sides at any measured location (all *p* = n.s.). However, the reduction in echogenicity relative to the contralateral side was significantly greater in the partial‐thickness group at 15, 30 and 45 mm proximal to the patella (15 mm: full −0.5 ± 10.9% vs. partial −21.4 ± 15.1%, *P* < 0.01; 30 mm: full 1.6 ± 10.4% vs. partial −11.6 ± 12.0%, *P* = 0.01; 45 mm: full −6.6 ± 5.0% vs. partial −16.0 ± 15.7%, *p* = 0.01). No significant difference was found at 60 mm (full −12.0 ± 8.5% vs. partial −16.3 ± 16.2%, *p* = n.s.).

The proportion of patients with reduced echogenicity did not differ significantly between the full‐ and partial‐thickness groups (1 out of 7 patients [14.3%] in the full‐thickness group vs. 9 out of 28 patients [32.1%] in the partial‐thickness group, *p* = n.s.). Tendon defects were observed in 6 out of 35 patients (17.1%) in the partial‐thickness group and 1 out of 7 patients (14.3%) in the full‐thickness group (*p* = n.s.).

## DISCUSSION

The most important finding of this study was that, at least 1 year after surgery, the QT morphology in patients who underwent QT autograft ACLR was comparable to that of the contralateral side throughout the entire harvest site. However, the A‐P thickness was slightly increased, and the echogenicity was significantly lower. A small defect at the harvest site was observed in 20% of patients, but there were no clinically significant complications related to the QT autograft harvest. Some patients displayed signs of vascularity on SMI evaluation, which was more frequently observed in the distal portion of the QT and more commonly in patients who were evaluated less than 2 years post‐operatively, indicating ongoing healing. Furthermore, the differences in thickness and echogenicity between the operated and contralateral sides were smaller in patients more than 2 years post‐operatively compared to those evaluated earlier. These findings suggest that the post‐operative QT undergoes a gradual normalization process beyond 1 year after surgery.

In this study, morphological healing, defect rates and residual vascularity of the QT did not differ between full‐ and partial‐thickness harvests. However, a greater reduction in echogenicity was observed in the partial thickness group. It is worth noting that the longer interval between ACLR and ultrasound evaluation in the full‐thickness group may have influenced these results.

A previous study evaluated the morphology of the QT and found that the harvested QT was slightly thicker and narrower; however, the assessment was limited to the distal half of the tendon (up to 30 mm proximal to the patella) [12]. In the current study, the proximal part of the tendon was also evaluated, which showed a thickening post‐operatively, while the distal part remained similar to the contralateral side. However, the mean change in thickness was only 1.3 mm (20%) at most, which suggests limited clinical significance. These findings indicate that the harvested QT undergoes morphological normalization at a minimum of 1 year post‐operatively.

Decreased echogenicity was observed in the harvested QT across all assessed locations, with mean differences exceeding the SEM of 2.0 at each site. Since echogenicity is influenced by collagen fibre density and organization, hypoechogenic changes are commonly seen in patients with tendinopathy [3, 9]. Additionally, a previous study using a rat Achilles tendon model demonstrated that echogenicity increased during the healing process and correlated with ultimate stress and stiffness of the tendon [4]. Therefore, the decreased echogenicity observed in the harvested QT may indicate that its structural properties have not yet fully recovered, particularly in the proximal portion of the tendon. However, an increase in tendon thickness may compensate for localized reductions in stiffness, and thus the overall stiffness of the tendon may not necessarily be inferior to that of an intact tendon. Notably, no complications related to QT harvest were observed in this study. The decreased echogenicity may reflect the tendon's reorganization process, though its relationship to quadriceps muscle recovery requires further investigation.

Although the feasibility of re‐harvesting the QT has not been directly investigated, some studies have examined re‐harvesting of the patellar tendon [15, 16, 19, 24]. A histological study reported increased cellularity and vascularity at the donor site [15], while another study found that tendon defects persisted even 6 years after ACLR, with the patellar tendon failing to return to a normal histological state [24]. The findings of persistent echogenicity alterations in the QT align with studies on the patellar tendon, suggesting that re‐harvesting the QT in revision ACLR may be challenging. However, further histological and biomechanical research on the harvested QT is necessary to determine its suitability for re‐harvesting.

Vascularity in the QT is a known healing response following tendon harvesting [5, 22]. In the current study, vascularity within the harvested tendon was observed in 29% of patients, whereas a previous study with a mean follow‐up of 6 months reported vascularity in 70% of patients [5]. Additionally, an ultrasound assessment using SMI at 2, 4, 6, 9 and 12 months after ACLR demonstrated that QT vascularity peaked at 4 months [22]. Considering these findings, along with the results of the current study showing that vascularity was more frequently observed in patients with a follow‐up of less than 2 years (58%) compared to those with more than 2 years (13%), it is likely that QT vascularity declines over time but is most commonly observed in the shorter follow‐up window (within the first 2 years) after ACLR.

A QT defect at the harvest site was observed in 20% of the included patients, which was comparable to a previous study with a minimum follow‐up of 2 years, reporting a 13% defect rate [12]. In contrast, a previous study using ultrasound to assess tendon defects 6 months after ACLR reported that 51 out of 61 patients (83.6%) had tendon defects, although the defect volume was small (7% of the total tendon volume) [5]. These findings suggest that the tendon defect may diminish over time. In this study, defects were more frequently observed at 15, 30 and 45 mm proximal to the patella than at 60 mm. This may be due to the lower tension in the proximal tendon when sutured, compared to the distal part near the patella. Additionally, blood flow from the surrounding muscle may have contributed to filling the defect. Notably, even in patients with tendon defects, no clinical complications were observed in this study.

This study has several limitations. First, the sample size was relatively small. While power analysis confirmed adequate statistical strength to detect differences in tendon thickness and echogenicity, it may have been insufficient for subgroup analyses—particularly for less common outcomes such as vascularity or comparisons between full‐ and partial‐thickness harvests. Second, the timing of post‐operative ultrasound assessments varied across participants, which may have influenced the evaluation of morphological recovery. Third, surgical techniques were not always consistent, particularly regarding harvest thickness and bone block inclusion, which limits the generalizability of the findings. Despite these limitations, this study provides valuable evidence that QT morphology is nearly restored at a minimum of 1 year following QT autograft harvest, although the persistently lower echogenicity suggests potential alterations in tendon quality.

## CONCLUSION

QT morphology following QT autograft ACLR was nearly normalized on ultrasound evaluation at a mean of 3.4 years post‐operatively. However, reduced echogenicity was observed, which may indicate compromised tendon quality. Despite this finding, no complications related to QT harvest were noted. Further histological and biomechanical studies are needed to assess the mechanical properties of the QT after harvest.

## AUTHOR CONTRIBUTIONS

All listed authors have contributed substantially to this work. Literature search, study design and primary manuscript preparation were performed by Jumpei Inoue, Kohei Kamada and Keishi Takaba. Data collection, analysis and interpretation of results were performed by Jumpei Inoue, Kohei Kamada, Joseph D. Giusto and M. Enes Kayaalp. Statistical analysis was performed by Jumpei Inoue and Kohei Kamada. Final manuscript drafting and editing were performed by Andrew L. Sprague, Bryson P. Lesniak and Volker Musahl.

## CONFLICT OF INTEREST STATEMENT

Volker Musahl reports educational grants, consulting fees and speaking fees from Smith & Nephew plc, educational grants from Arthrex and DePuy/Synthes, is a board member of the *International Society of Arthroscopy, Knee Surgery and Orthopaedic Sports Medicine* (ISAKOS), and deputy editor‐in‐chief of *Knee Surgery, Sports Traumatology, Arthroscopy* (KSSTA). The remaining authors declare no conflicts of interest.

## ETHICS STATEMENT

This study was approved by the institutional review board (IRB), that is, the Ethics Committee of the University of Pittsburgh, in Pittsburgh, USA (reference ID no. STUDY 23040125). Written informed consent was obtained from all participants.

## Supporting information

STROBE‐checklist‐v4‐combined‐PlosMedicine.

## Data Availability

Anonymised data from the study are available upon reasonable request.
